# Histone demethylase PHF2 regulates inflammatory genes in Alzheimer’s disease

**DOI:** 10.1038/s41380-025-03181-z

**Published:** 2025-08-23

**Authors:** Guojun Yang, Yong Ren, Ping Zhong, Prechetas Jai Patel, Xiao-Qing Chen, Lei Wan, Young-Ho Lee, Komal Saleem, Jian Feng, Zhen Yan

**Affiliations:** Department of Physiology and Biophysics, State University of New York at Buffalo, Jacobs School of Medicine and Biomedical Sciences, Buffalo, NY USA

**Keywords:** Diseases, Neuroscience

## Abstract

A plethora of factors contribute to cognitive impairment in Alzheimer’s disease (AD), including neuroinflammation, synaptic dysfunction and gene alteration. In search of transcription factors controlling dysregulated genes in AD, we identified that the histone demethylase PHF2 (KDM7C) was a top-ranking candidate. Significant upregulation of PHF2 was found in AD human postmortem tissues, iPSC-derived neurons from AD patients, and a familial AD mouse model (5xFAD). ChIP-seq analysis and quantitative PCR profiling with bidirectional manipulation of Phf2 revealed that Phf2 regulated many genes critically involved in inflammatory pathways and neurodegeneration, including *Stat3, Nfkbia, Nfkb2, Tnfrsf1a, Fgfr1, IL6st, Notch2*, and *Csf1*. Knockdown of Phf2 in 5xFAD mice reduced the expression of inflammatory genes, leading to the substantial reduction of microglia/astrocyte activation and the restoration of glutamatergic synaptic function. Behavioral studies showed that Phf2 knockdown in 5xFAD mice significantly improved performance in the Barnes maze test, indicating a mitigation of spatial memory deficits. Our findings have revealed the epigenetic enzyme PHF2 as a regulator of neuroinflammatory processes in AD, linking its activity to both gene expression and cognitive outcomes. It suggests that targeting PHF2 could be a novel therapeutic approach for AD and other brain disorders involving neuroinflammation.

## Introduction

Neuroinflammation has emerged as a critical factor in the pathophysiology of Alzheimer’s disease (AD), a neurodegenerative disorder characterized by significant cognitive decline and memory loss [1–3]. Chronic activation of the brain’s immune system leads to the release of pro-inflammatory cytokines and other inflammatory mediators [4, 5]. This persistent inflammatory state is thought to contribute to neuronal damage, directly impacting cognitive brain regions during AD progression [3, 6]. Understanding the regulatory mechanisms that control the expression of inflammatory genes in the brain is important for identifying potential therapeutic targets to alleviate memory deficits.

Epigenetic alterations, including changes in DNA methylation and histone modification, have emerged as a significant player in aging [7] and AD [8]. Epigenome-wide association studies of AD patients identified many differentially methylated regions including those containing AD susceptibility variants [9, 10]. Losses and gains of H4K16ac, H3K27ac and H3K9ac peaks at different loci were also found from ChIP-seq profiling of AD brains, which was linked to transcriptomic changes [11–13]. Preclinical studies demonstrated that inhibition of histone deacetylases or histone methyltransferases mitigated AD pathology, restored synaptic genes and ameliorated cognitive impairment in AD models [14–19].

In search of transcription factors controlling dysregulated genes in AD, we identified PHF2 (plant homeodomain finger 2) as a top-ranking candidate. PHF2 (also known as KDM7C) is a histone demethylase with roles in cellular processes, such as chromatin organization, DNA replication, DNA repair, RNA polymerases I and II gene transcription [20–32]. PHF2 regulates gene expression through epigenetic modifications in both demethylase-dependent and independent mechanisms [22, 26]. After activation by protein kinase A phosphorylation, PHF2 forms a complex with and demethylates ARID5B, subsequently removing dimethylated histone H3 lysine 9 (H3K9me2) to activate gene transcription [29, 33, 34]. In macrophages, NF-κB-dependent delivery of PHF2 to a subset of inflammatory response genes triggers their activation by the erasure of trimethylated histone H4 lysine 20 (H4K20me3) [35]. PHF2 has been implicated in various diseases, including non-alcoholic fatty liver, autism spectrum disorder and cancer [34, 36–38]. However, the role of PHF2 in AD-related neuroinflammation and memory loss remains largely unexplored.

In this study, we investigated the alteration of PHF2 expression and its consequence in AD. We provide evidence showing that PHF2 is a regulator of inflammatory gene expression in AD and its upregulation contributes to the neuroinflammatory processes underlying the disease. Importantly, we show that PHF2 knockdown not only reduces inflammation, but also improves synaptic function and cognitive behavior in a familial AD mouse model. These findings open new avenues for the development of epigenetic-based therapies aimed at modulating PHF2 activity to mitigate AD phenotypes.

## Results

### PHF2 expression is significantly increased in AD humans and an AD mouse model

**Fig. 1:**
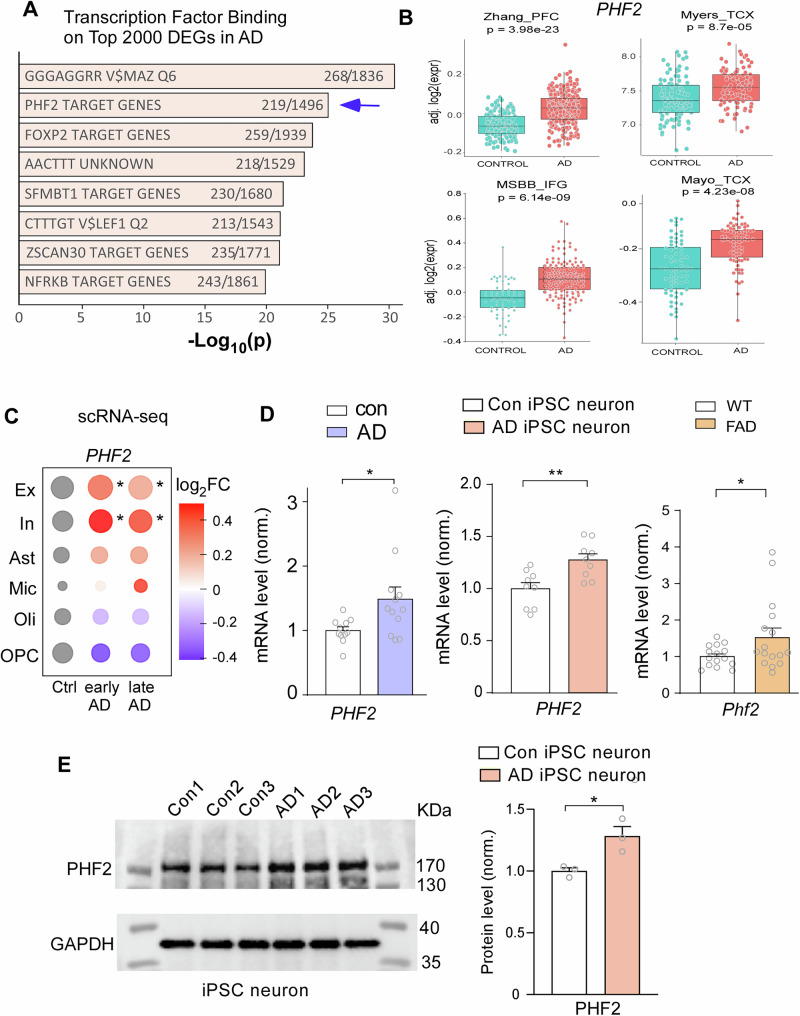
*PHF*2 expression is increased in AD humans and 5xFAD mice. **A** Enrichment of transcription factor (TF) binding sites on top 2000 human AD DEGs [39]. X-axis, negative Log_10_ value of probability. Also shown are Genes from Input / Genes in Annotation to reflect the number of detected AD DEGs among target genes for each TF. **B** Box plots showing *PHF*2 mRNA levels in human postmortem brain tissues (PFC, TCX and IFG) obtained from microarray and RNAseq studies [40–43]. *p* values were from Wilcoxon Rank-Sum tests. **C** Heat maps from a scRNA-seq dataset [44] showing PHF2 expression changes in different cell types in AD humans with early or late pathology, compared to controls. Circle size reflects PHF2 expression levels. *, *P*_adj._ < 0.05. Ex: excitatory neuron; In: inhibitory neuron; Ast: astrocyte; Mic: microglia; Oli: oligodendrocyte; OPC: oligodendrocyte precursor cell. **D** Bar graphs showing PHF2 mRNA levels in human postmortem PFC of control (*n* = 11) vs. AD (*n* = 12), iPSC-derived cortical neurons from control (*n* = 9 cultures/3 lines from 3 subjects) vs. AD patients (*n* = 9 cultures/3 lines from 3 subjects), and mouse PFC from 6–8 months old WT (*n* = 16) vs. 5xFAD (*n* = 16). **E** Western blots and bar graphs of PHF2 proteins in iPSC-derived cortical neurons from control vs. AD patients (*n* = 3 lines from 3 subjects/group). Full-length PHF2 (top band) intensity was normalized against GAPDH in quantitation. Data are presented as mean ± SEM. *p < 0.05, **p < 0.01, unpaired t-test.

AD has many differentially expressed genes (DEGs). To find out the master regulators controlling these DEGs, we used ToppGene to examine the transcription factors with binding sites on top 2000 AD DEGs ranked by transcriptomics [39]. PHF2 was identified as a top-ranking transcription factor (*p* = 4.47e–25) with 219 AD DEGs as its targets (Fig. 1A and Supplementary Table 1), suggesting that PHF2 is a key player involved in gene dysregulation in AD.

We next investigated potential alterations of PHF2 expression in AD using large-scale datasets of human postmortem brain tissues from AD patients and control subjects. PHF2 expression was significantly increased in prefrontal cortex (PFC) and temporal cortex (TCX) of AD in two microarray studies [40, 41] (Fig. 1B, Zhang, *p* = 3.98e–23; Myers, *p* = 8.7e–05, Wilcoxon Rank-Sum test). PHF2 expression was also significantly increased in inferior frontal gyrus (IFG) and TCX of AD in two RNAseq studies [42, 43] (Fig. 1B and Supplementary Table 2, MSBB, syn27068762, *p* = 6.14e–09; Mayo, syn27024969, *p* = 4.23e–08, Wilcoxon Rank-Sum test). More detailed analysis of 3 bulk RNAseq datasets (Mayo, MSBB, ROSMAP) further showed that PHF2 was significantly increased in both sexes of AD samples, and most prominent increase was in people with severe AD (Braak stage ≥ 4) (Supplementary Fig. 1 and Supplementary Table 3).

To gain insights into the cell types with altered PHF2 expression in AD, we examined the single-cell RNA-seq dataset [44]. As shown in Fig. 1C, compared to control samples (‘no-pathology’), PHF2 was significantly increased in excitatory neurons (Ex) and inhibitory neurons (In) of the ‘early-pathology’ AD samples (Ex: *p* = 5.08E–12; In: *p* = 0.0097), as well as the pooled AD samples (‘early pathology’ and ‘late-pathology’) (Ex: *p* = 3.83E–07; In: *p* = 0.0198). PHF2 also showed the trend of increase in astrocytes and microglia of AD samples, but did not reach the statistical significance (Supplementary Table 4).

To validate the increase of PHF2 expression in AD, we performed qPCR analyses with mRNA extracted from human postmortem PFC, a key cognitive region impaired in AD. We found that PHF2 expression in AD patients was 44.7 ± 17.7% higher than in controls (Fig. 1D, *p* = 0.027, t-test). Using human iPSC-derived cortical neurons (D40), we found that PHF2 expression level was 27.7 ± 6.1% higher in AD patients than in controls (Fig. 1D, *p* = 0.0042, t-test). Moreover, PHF2 mRNA level was 66.1 ± 26.4% higher in the PFC of 5xFAD mice (6–8 months old), compared to age-matched wild-type controls (Fig. 1D, *p* = 0.037, t-test). Western blot analysis of iPSC-derived cortical neurons confirmed the significant increase of PHF2 protein levels in AD samples (Fig. 1E, *p* = 0.025, t-test).

These findings consistently demonstrate the elevated PHF2 expression in AD across various models. As PHF2 is an epigenetic regulator, its increase in AD could significantly influence the expression of downstream genes.

### PHF2 targets in AD DEGs are enriched in inflammation genes

**Fig. 2:**
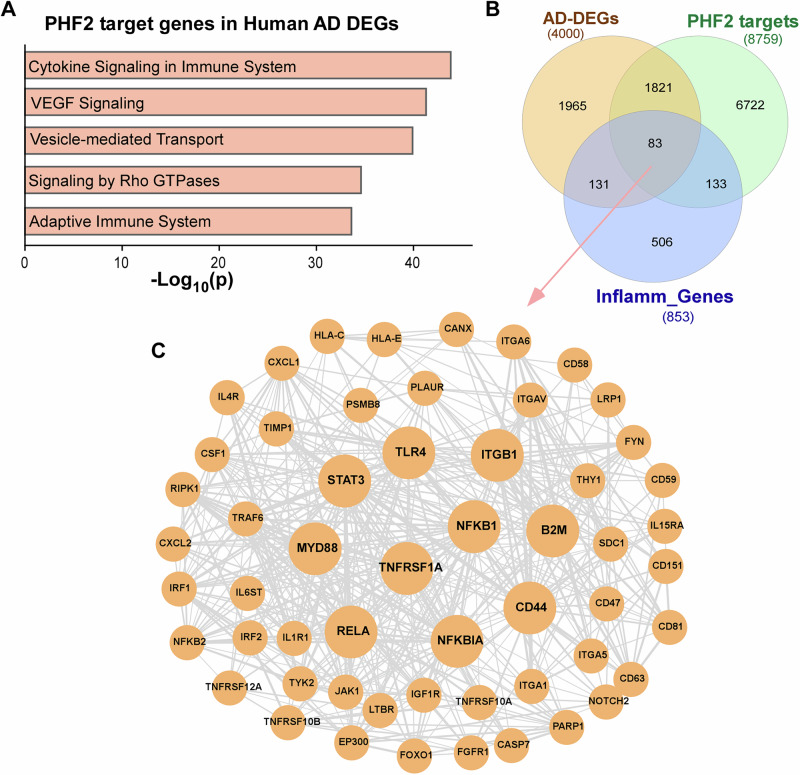
*PHF2* targets in AD DEGs are enriched in immune response genes. **A** Gene ontology (GO) enriched pathways of the commons genes from PHF2 ChIPseq targets and human AD DEGs. **B** Venn diagram showing the overlap of inflammation genes (blue), human AD DEGs (yellow), and PHF2 target genes (green). **C** Protein-protein interaction (PPI) network of the overlapping genes in **B**. Hub genes are shown in large circles.

To understand how the increased expression of PHF2 may influence AD pathophysiology, we investigated its impact on the expression of genes involved in the disease. ChIP-seq data from ChIP-Atlas have revealed 8759 genes with PHF2 binding on their promoters. These PHF2 targets and AD DEGs (4000, combined from top 2000 DEGs in a RNAseq dataset [39] and 2577 DEGs in a microarray dataset [40]) have 1904 overlapped genes. Gene ontology (GO) enrichment analyses of these PHF2 targets within AD DEGs (1904) revealed a significant enrichment of genes associated with cytokine signaling and adaptive immune system (Fig. 2A), suggesting that PHF2 may play a role in modulating inflammatory pathways.

Given that chronic neuroinflammation is a hallmark of human AD [3], we sought to identify the genes potentially regulated by PHF2 that contribute to neuroinflammation. We discovered 83 genes that were common among inflammation-related genes (853), PHF2 target genes (8759) and AD DEGs (4000) (Fig. 2B and Supplementary Table 5). Among these common genes, 78 form a closely connected gene network (Fig. 2C) with *STAT3, CD44, NFKB1, TNFRSF1A, TLR4, NFKBIA, B2M, ITGB1, MYD88* and *RELA* as hub genes (>25 edges). Many of the genes in the network, such as *NOTCH2, STAT3, CSF1, RELA* and *FOXO1*, regulate the expression of numerous other genes. Consequently, changes in their expression can lead to a significant alteration of the overall transcriptome landscape in AD. These findings underscore the regulatory role of PHF2 in neuroinflammatory processes and point to PHF2 as a potential therapeutic target for modulating inflammation associated with AD.

### Phf2 regulates the expression of AD-associated inflammation genes

**Fig. 3:**
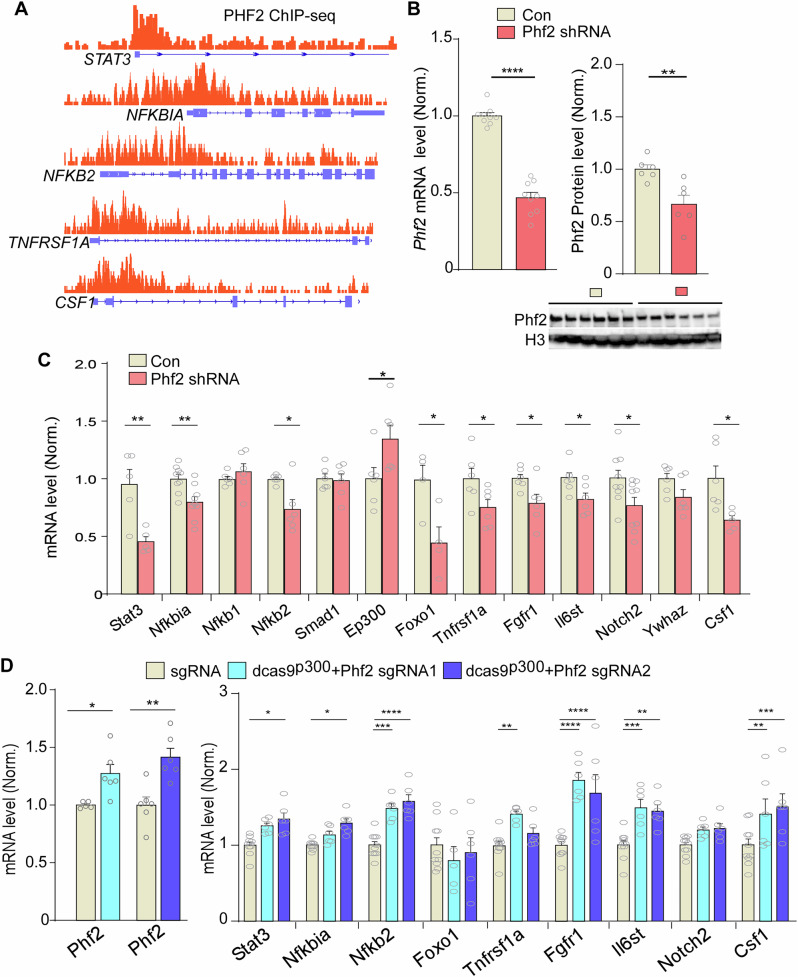
Phf2 knockdown or overexpression bi-directionally affects the expression of AD-related inflammation genes. **A** PHF2 ChIPseq landscapes on representative inflammation genes in a human cell line (NAMEC8). **B** Bar graphs of qPCR data showing Phf2 mRNA and protein levels in N2A cells transfected with control or Phf2 shRNA plasmids (qPCR: *n* = 9 cultures/group; WB: *n* = 6 cultures/group). Inset: Western blot images of Phf2 protein extracted from nuclear fraction of transfected N2A cells. Phf2 was normalized to histone 3 in quantification. **C** Bar graphs of qPCR data showing mRNA levels of representative inflammation genes that are PHF2 targets among AD DEGs in N2A cells transfected with control or Phf2 shRNA plasmids (*n* = 4–9 cultures/group). **D** Bar graphs of qPCR data showing mRNA levels of Phf2 and its targets in N2A cells transfected with dCas9-p300 plus Phf2 sgRNA (sgRNA1 or sgRNA2) or sgRNA control (*n* = 5–6/group). All data are presented as mean ± SEM. *p < 0.05, **p < 0.01, ***p < 0.001, unpaired t-test **B,** **C** or one-way ANOVA with post-hoc Tukey’s multiple comparisons **D**.

Next, we investigated whether the expression of AD-related inflammation genes is directly regulated by PHF2. We selected genes from the aforementioned network (Fig. 2C). ChIP-seq landscapes show the strong occupancy of PHF2 around the transcription start sites (TSS) of these genes (Fig. 3A).

We first examined the effect of Phf2 knockdown on these genes. N2A cells transfected with a Phf2 short hairpin RNA (shRNA) showed a 53.3 ± 4.2% (*p* < 0.0001, t-test) reduction of Phf2 mRNA (Fig. 3B) and a 42.5 ± 6.0% (*p* = 0.006, t-test) reduction of Phf2 protein in the nucleus (Fig. 3B), confirming the effectiveness of Phf2 knockdown. Quantitative PCR revealed a significant reduction of most of these AD-related inflammation genes by Phf2 knockdown (Stat3: 52.1 ± 7.9%, *p* < 0.01, Nfkbia: 20.5 ± 1.4%, *p* < 0.01, Nfkb2: 26.5 ± 2.9%, Foxo1: 55.1 ± 16.4%, Tnfrsf1a: 24.8 ± 2.9%, Fgfr1: 21.3 ± 2.0%, IL6st: 17.8 ± 1.4%, Notch2: 23.1 ± 2.6%, Csf1: 36.0 ± 4.2%, all *p* < 0.05) (Fig. 3C).

On the other hand, we examined the effect of Phf2 overexpression on AD-related inflammation genes. The CRISPR-based epigenome editing approach was used to recruit p300 to Phf2 promoter regions with specific single guide RNA (sgRNA), therefore increasing its acetylation and expression [45]. N2A cells transfected with dCas9-p300 and one sgRNA against *Phf2* promoter showed significantly increased Phf2 mRNA levels (sgRNA1: 31.5 ± 1.9%, *p* = 0.01, sgRNA2: 41.7 ± 3.8%, *p* = 0.003, t-test), compared to those transfected with sgRNA alone (Fig. 3D). Among the 9 genes downregulated by Phf2 knockdown, 7 genes were significantly upregulated by Phf2 overexpression to various extents (Fig. 3D, 29–86%, F_2,170_ = 46.56, *p* < 0.001 to *p* = 0.02, one-way ANOVA). The bi-directional evidence for the majority of these genes indicates that AD-related inflammation genes can be directly regulated by Phf2.

**Fig. 4:**
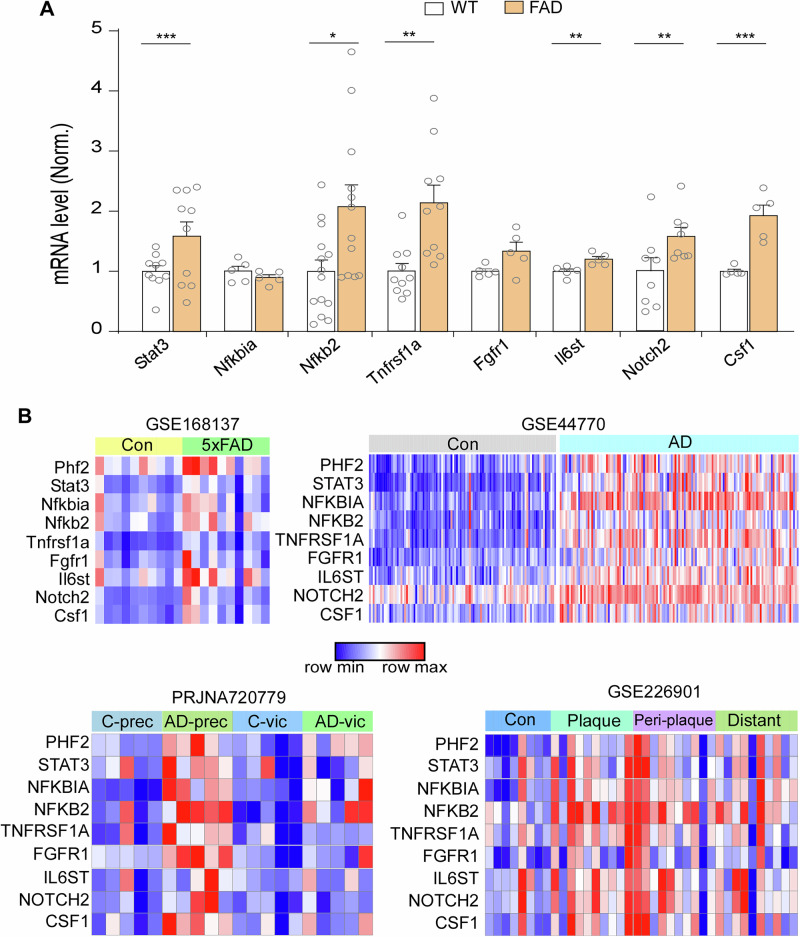
Phf2 targeted inflammation genes are increased in 5xFAD mice and AD patients. **A** Bar graphs of qPCR data showing mRNA levels of Phf2 targeted inflammation genes in PFC of WT vs. 5xFAD mice (*n* = 5–14/group). All data are presented as mean ± SEM. *p < 0.05, **p < 0.01, ***p < 0.001, unpaired t-test. **B** Heat map illustrating the expression levels of Phf2 and its inflammation-related target genes in an RNAseq dataset (GSE168137) [46] comparing cortex of 4-month-old WT vs. 5xFAD mice, a human microarray dataset (GSE44770) [40] from postmortem PFC of control vs. AD, a Bioproject dataset (PRJNA720779) [47] comparing precuneus (prec) and visual (vic) cortex of control vs. AD patients, and an RNAseq dataset (GSE226901) [48] comparing microdissected temporal cortex from control vs. AD samples at different distances to β-amyloid plaques. The color scheme ranges from blue to red, corresponding to changes in gene expression levels, with blue indicating a decrease and red indicating an increase.

Given the increased Phf2 expression in AD humans and 5xFAD mice (Fig. 1), we investigated whether the expression of Phf2 targets involved in inflammation was also changed in AD. Our qPCR profiling revealed the significant increase of proinflammatory genes, such as *Stat3, Nfkb2, Tnfraf1a, IL6st, Notch2* and *Csf1*, in PFC of 5xFAD mice (Fig. 4A, 20–114%, *p* < 0.05 to *p* < 0.001, t-test). Similar increases of Phf2 and its inflammation-related target genes were also observed in the cortex of 5xFAD (4 months old) mice from an RNAseq dataset (GSE168137) [46] (Fig. 4B) and in AD human postmortem tissues from a microarray study (GSE44770) [40] (Fig. 4B). Such increases were region specific, as a profound increase was found in precuneus cortex, but not in visual cortex, of AD patients from an RNAseq dataset (PRJNA720779) [47] (Fig. 4B). Moreover, based on an RNAseq dataset about transcriptomic responses to Aβ plaques and tau tangles in AD (GSE226901) [48], the biggest increase of Phf2 and its inflammation-related target genes was found in peri-plaque and plague regions (Fig. 4B). Taken together, these results suggest that the elevated expression of inflammation genes in AD may be caused by upregulated Phf2 in AD.

**Fig. 5:**
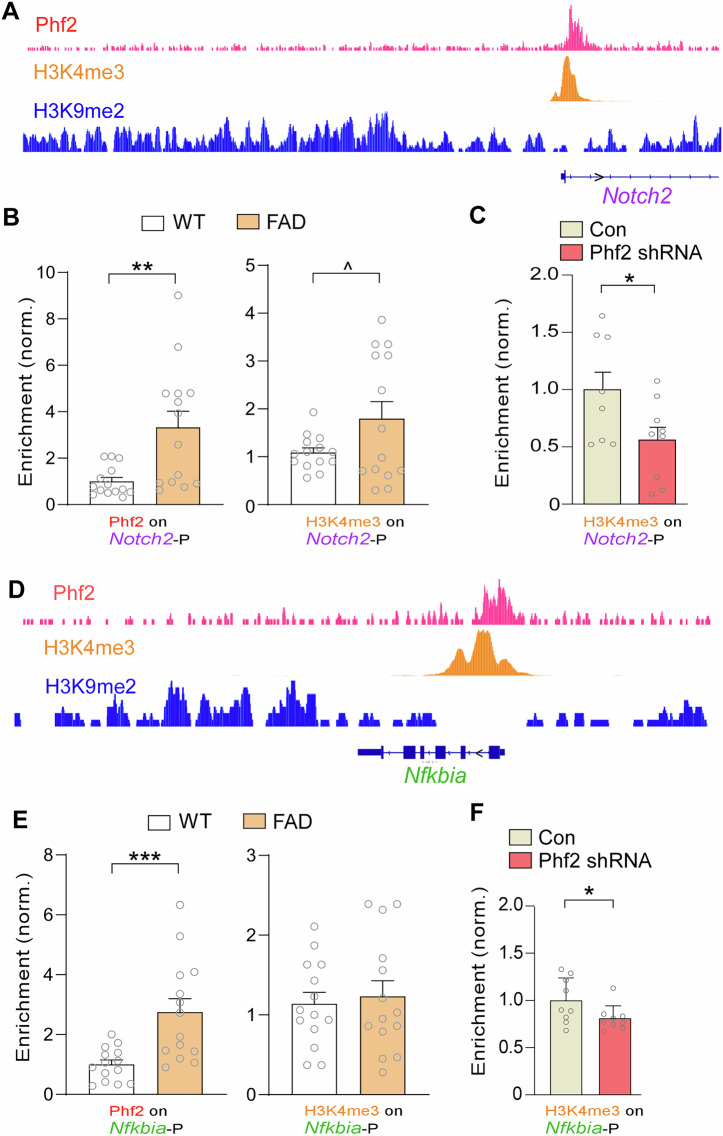
Phf2 regulates inflammation gene expression via a mechanism involving H3K4me3. **A,** **D** Mouse Phf2 (SRX4991505, neural stem cell), H3K4me3 and H3K9me2 ChIPseq landscapes on representative inflammation genes (**A**: *Notch2*, **D**: *Nfkbia*) in mouse tissue. **B,** **E** Bar graphs of ChIP-PCR data showing Phf2 and H3K4me3 occupancy at the promoter (P) region of *Notch2* (**B**) or *Nfkbia* (E) in PFC of WT vs. 5xFAD mice (*n* = 7 mice/group, 2 technical repeats per sample). **C,** **F** Bar graphs of ChIP-PCR data showing H3K4me3 occupancy at *Notch2* (**C**) or *Nfkbia* (**F**) promoter in N2A cells transfected with control vs. Phf2 shRNA (*n* = 3 cultures from 3 independent experiments/group, 3 technical repeats per sample). Data are presented as mean ± SEM. ^*p* < 0.1, *p ≤ 0.05, **p ≤ 0.01, ***p ≤ 0.001, unpaired t-test.

PHF2 is considered as a H3K4me2/3 reader and H3K9me2/3 eraser [21, 49, 50]. Based on mouse ChIPseq landscapes, Phf2 occupancy largely overlaps with H3K4me3 peaks at the promoter region of inflammation genes (e.g. Notch2, Nfkbia), while H3K9me2 has no enrichment at these loci (Fig. 5A, D). To find out the potential mechanism underlying Phf2 regulation of inflammation genes, we performed ChIP-PCR to examine alterations of Phf2 or H3K4me3 occupancy at their promoters in PFC of 5xFAD mice (with increased Phf2 expression) and in N2A cells transfected with Phf2 shRNA (with decreased Phf2 expression).

Phf2 and H3K4me3 occupancy at *Notch2* promoter was substantially increased in 5xFAD mice (Fig. 5B, Phf2: 232.8%, *p* = 0.003; H3K4me3: 64.9%, *p* = 0.07, t-test). On the other hand, H3K4me3 occupancy at *Notch2* promoter was significantly decreased by Phf2 knockdown (Fig. 5C, 44%, *p* = 0.04, t-test). Phf2 occupancy at *Nfkbia* promoter was significantly increased in 5xFAD mice, while H3K4me3 occupancy was unchanged (Fig. 5E, Phf2: 174.9%, *p* = 0.001; H3K4me3: 8.2%, *p* = 0.7, t-test), which may be due to the small genome area covered by ChIP assay. However, H3K4me3 occupancy at *Nfkbia* promoter was significantly decreased by Phf2 knockdown (Fig. 5F, 20%, *p* = 0.05, t-test). These data suggest that Phf2 may regulate inflammatory gene expression via altering H3K4me3, a histone mark linked to gene activation.

### Phf2 knockdown attenuates inflammation gene expression and microglia/astrocyte activation in 5xFAD mice

**Fig. 6:**
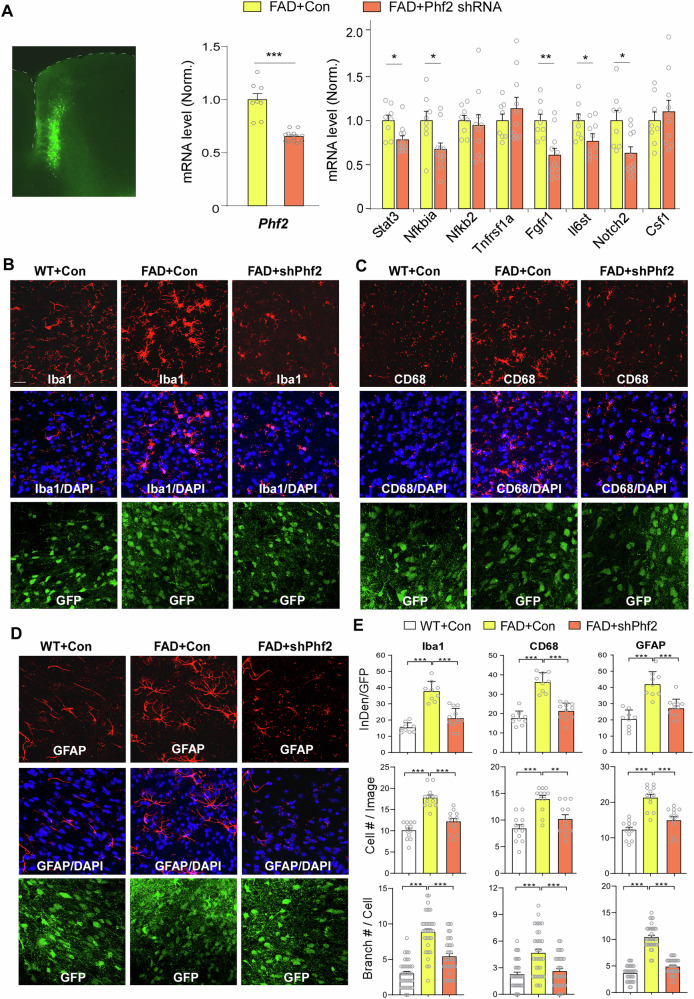
Phf2 knockdown in 5xFAD mice reduces inflammation gene expression and microglial/astrocyte activation. **A** Bar graphs of qPCR data showing mRNA levels of Phf2 and Phf2 targeted inflammation genes in PFC of 5xFAD mice injected with control vs. Phf2 shRNA AAV (*n* = 8–12/group). Inset: a confocal image showing the viral-infected PFC region from a mouse with the stereotaxic injection of Phf2 shRNA AAV (GFP-tagged). **B**–**D** Confocal microscopy images of Iba1 **B**, CD68 **C** and GFAP **D** staining of PFC slices from WT vs. 5xFAD mice injected with GFP control (Con) or Phf2 shRNA (shPhf2) AAV. **E** Bar graphs showing the integrated intensity of Iba1, CD68 or GFAP signals (normalized to GFP), the number of Iba1 + , CD68+ or GFAP+ cells per image, and the average number of branches per cell positive for Iba1, CD68 or GFAP, in prelimbic cortex (a subregion of medial PFC) of different groups. Data were obtained from 9–12 images of 9 slices of 3 mice per group. Data are presented as mean ± SEM. *p < 0.05, **p < 0.01, ***p < 0.001, unpaired t-test **A** or one-way ANOVA with post-hoc Tukey’s multiple comparisons **E**.

With the elevation of Phf2 in AD, we next tested whether Phf2 knockdown in 5xFAD mice could alleviate pathological phenotypes. Mice with PFC injection of Phf2 shRNA AAV showed a significant reduction of Phf2 mRNA (Fig. 6A, 35 ± 6%, *p* < 0.0001, t-test), confirming the in vivo knockdown effectiveness. The expression of inflammatory genes, such as *Stat3, Nfkbia, Fgfr1, IL6st* and *Notch2*, was significantly reduced in Phf2 shRNA-injected 5xFAD mice (Fig. 6A, 21–40%, *p* < 0.05 to *p* < 0.01, t-test), suggesting that the elevated inflammation genes in AD can be reversed by Phf2 knockdown.

Knockdown of PHF2 in human neural stem cells led to the transcriptional alteration of many genes (increase of 791 genes and decrease of 938 genes) [23], highlighting the complex influence of PHF2 on gene expression. To find out whether other genes were also affected by Phf2 knockdown in 5xFAD mice, we profiled a few synaptic genes encoding proteins at glutamatergic or GABAergic synapses. As shown in Supplementary Fig. 2, Phf2 knockdown in 5xFAD mice did not induce significant changes on most of the synaptic genes, including Gria2, Stxbp5, Gad1, and Gabra4.

To assess the overall impact of Phf2 knockdown on brain inflammation, we examined the state of glial cells by staining PFC slices with microglial markers Iba1 and CD68, as well as the astrocyte marker GFAP. Iba1-stained microglial cells (Fig. 6B) exhibited barely visible cell bodies with long, thin processes in WT mice injected with control shRNA AAV, indicating a resting state within a non-inflammatory environment, however, they displayed enlarged cell bodies with thick, short processes in 5xFAD mice injected with control shRNA AAV, suggesting an activated state indicative of ongoing inflammation or tissue damage. In 5xFAD mice injected with Phf2 shRNA AAV, there was a marked reduction of microglial cell body size, the number of cells with visible cell bodies, and process thickness, indicating a less activated state and an attenuated inflammatory response. A similar pattern was observed with the staining of CD68 (an active microglia marker), suggesting a reduced phagocytic activity of microglia in AD by Phf2 knockdown (Fig. 6C).

Similarly, GFAP-stained astrocytes (Fig. 6D) showed significantly higher GFAP levels in 5xFAD mice than in WT mice, indicating astrocyte activation and increased inflammation. 5xFAD mice injected with Phf2 shRNA AAV exhibited strongly reduced GFAP levels, suggesting decreased astrocyte activation and inflammation.

To quantify the changes in these markers, we compared the signal intensities, cell densities and branch numbers per cell positive for Iba1, CD68, or GFAP. As shown in Fig. 6E, compared to control AAV-injected WT mice, a significant increase of the three parameters for the three markers were detected in control AAV-injected 5xFAD mice, which was significantly reversed in 5xFAD mice injected with Phf2 shRNA AAV (Iba1: F_2,27_ = 41.9 (intensity), F_2,33_ = 35.6 (density), F_2,105_ = 53.6 (branch#); CD68: F_2,27_ = 49.2 (intensity), F_2,33_ = 14.2 (density), F_2,105_ = 15.5 (branch#); GFAP: F_2,27_ = 26.8 (intensity), F_2,33_ = 24.6 (density), F_2,105_ = 167.0 (branch#), all *p* < 0.01 or *p* < 0.001, one-way ANOVA). These findings suggest that Phf2 knockdown attenuates microglial and astrocyte activation in an AD model, which could reduce chronic inflammation.

### Phf2 knockdown restores synaptic function and improves spatial memory in 5xFAD mice

**Fig. 7:**
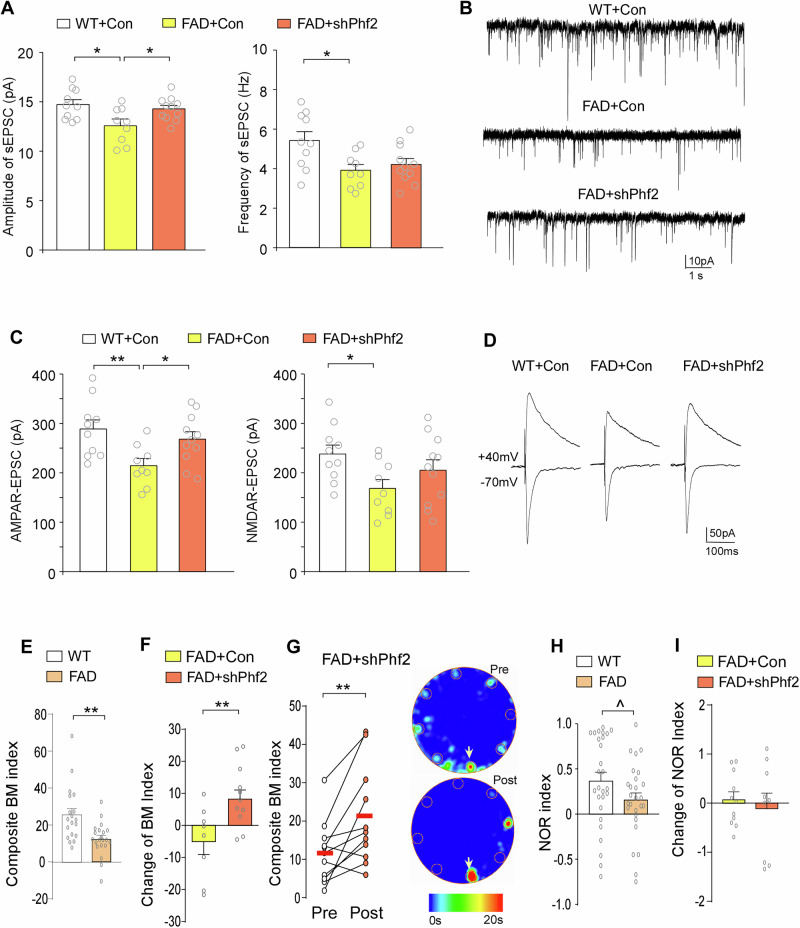
Phf2 knockdown in 5xFAD mice restores synaptic function and improves spatial memory. **A** Bar graphs of the amplitude and frequency of spontaneous EPSC (sEPSC) in GFP-positive PFC pyramidal neurons from WT vs. 5xFAD mice (6-month-old) with PFC injection of control (Con) or Phf2 shRNA (shPhf2) AAV (*n* = 9–11 cells/3–4 mice/group). **B** Representative sEPSC traces in each group. **C** Bar graphs of AMPAR-EPSC or NMDAR-EPSC amplitudes evoked by an electrical stimulation in the same groups of neurons (*n* = 9–11 cells/3–4 mice/group). **D** Representative traces of evoked EPSC in each group. **E** Bar graphs of spatial memory index in Barnes maze (BM) tests of WT vs. 5xFAD mice (6–7 months old, *n* = 19–21 mice/group). **F** Bar graphs showing changes in spatial memory index of 5xFAD mice with PFC injection of control (Con) or Phf2 shRNA (shPhf2) AAV (*n* = 8–11 mice/group). **G** Chart of spatial memory index of individual 5xFAD mice before (Pre) and 1-month after (Post) PFC injection of Phf2 shRNA AAV (*n* = 11 mice). Inset, Representative heat maps of BM tests of a 5xFAD mouse showing the time spent at various locations in the memory phase before (Pre) and after (Post) injection of Phf2 shRNA AAV. Red circles: holes on the platform; Arrowheads point to correct holes. **H** Bar graphs of discrimination ratio in the novel object recognition (NOR) tests of WT vs. 5xFAD mice (6-month-old, *n* = 21 mice/group). **I** Bar graphs showing changes in NOR discrimination ratio of 5xFAD mice with PFC injection of control (Con) or Phf2 shRNA (shPhf2) AAV (*n* = 9–11 mice/group). ^*p* < 0.1, *p < 0.05, **p < 0.01, one-way ANOVA with post-hoc Tukey’s multiple comparisons (**A,** **C**) or t-test (unpaired: **E,** **F,** **H**; paired: **G**).

Microglia/astrocyte activation and neuroinflammation in AD could lead to synaptic dysfunction through synapse elimination or modulation [51]. To evaluate the impact of Phf2 knockdown in 5xFAD mice on synaptic function, we measured synaptic currents in PFC pyramidal neurons. As shown in Fig. 7A, B, in 5xFAD mice injected with control AAV, both the amplitude and the frequency of spontaneous excitatory postsynaptic currents (sEPSC) were significantly reduced, compared to WT mice injected with control AAV (Amp: 17% reduction, Freq: 28% reduction). However, in 5xFAD mice injected with Phf2 shRNA AAV, the amplitude of sEPSC was significantly elevated (F_2,27_ = 4.7, *p* = 0.012, one-way ANOVA).

We further measured AMPAR- and NMDAR-mediated EPSC evoked by electrical stimulations (Fig. 7C, D). Compared to WT, the significantly reduced AMPAR-EPSC amplitude (26%) and NMDAR-EPSC amplitude (29%) was detected in 5xFAD mice. Injection of Phf2 shRNA AAV to 5xFAD mice significantly elevated AMPAR-EPSC amplitude in PFC neurons (F_2,27_ = 4.4, *p* = 0.02, one-way ANOVA), while a trend of increase was also observed with NMDAR-EPSC amplitude. These findings suggest that Phf2 knockdown in 5xFAD mice largely restored the diminished synaptic function in PFC pyramidal neurons, particularly AMPAR function.

Synaptic dysfunction is strongly associated with cognitive impairment in AD. With the restoration of synaptic function by Phf2 knockdown in 5xFAD mice, we next investigated the impact of Phf2 knockdown on cognitive behaviors in this AD model. Barnes Maze, which tests the animal’s ability of recalling the location of one correct hole (where an escape box was attached before the test phase) from 7 other incorrect holes on a round platform, was used to examine spatial memory. The spatial memory index was calculated with a formula considering the time spent on exploring the correct hole and incorrect holes and the latency of finding the correct hole. As shown in Fig. 7E, 5xFAD mice had significantly lower spatial memory index than wild-type mice (−51.4 ± 15.1%, *p* = 0.033, t-test). To investigate the effect of Phf2 knockdown on spatial memory loss, Barnes Maze tests were conducted on 5xFAD mice before and 1-month after stereotaxic injection of Phf2 shRNA AAV to the PFC. Improvement in spatial memory was measured by the increase of post-injection index compared to pre-injection index. As shown in Fig. 7F, 5xFAD mice injected with control vs. Phf2 shRNA AAV had significantly different changes in spatial memory index (control: −5 ± 4, Phf2 shRNA: 9.4 ± 2.9, *p* = 0.008, t-test). A significant increase of spatial memory index was observed after Phf2 shRNA AAV injection in individual 5xFAD mice (Fig. 7G, *p* = 0.009, paired t-test). As shown in the representative heatmaps, 5xFAD mice spent significantly more time at the correct hole post-injection of Phf2 shRNA AAV than pre-injection. These results suggest that Phf2 knockdown in the PFC can improve spatial memory in 5xFAD mice.

We also performed novel object recognition (NOR) tests on these mice. While the discrimination ratio was decreased in 5xFAD mice, compared to WT counterparts (Fig. 7H, *p* = 0.076, t-test), 5xFAD mice with PFC injection of Phf2 shRNA AAV did not improve NOR performance, compared to those injected with control AAV (Fig. 7I, *p* = 0.6, t-test).

## Discussion

In this study, we have identified the histone demethylase PHF2 (KDM7C) as an important upstream regulator of inflammatory genes in AD, which contributes to synaptic dysfunction and memory deficits of the disease. We demonstrated that PHF2 expression was upregulated in AD across multiple models, including human postmortem tissues, iPSC-derived neurons, and 5xFAD mice. In addition to transcriptomic evidence, PHF2 was ranked high in phosphoproteomic and genome-wide association studies of AD [39], further supporting its involvement in the disease. Further research is needed to explore how PHF2 is elevated in AD.

One of our key findings is that PHF2 regulates the expression of critical inflammation-related genes, such as Stat3, Nfkbia, Nfkb2, Tnfrsf1a, Fgfr1, IL6st, Notch2, and Csf1. The increased expression of these genes in AD tissues highlights the regulatory influence of PHF2 on neuroinflammatory processes. In the 5xFAD mouse model, knockdown of Phf2 led to a significant reduction of these genes, further confirming the role of PHF2 in modulating inflammatory gene expression. Regulation of Stat3 and IL6st by Phf2 is particularly interesting, as these two genes are at the critical control points of the IL11-induced canonical STAT3 and non-canonical ERK-mTORC1 signaling modules [52–55]. It has been reported that inhibition of IL11 results in remarkable increases in both healthspan and lifespan of mice [56]. Therefore, a reduced level of Stat3 and IL6st by PHF2 knockdown in AD could have beneficial effects on healthspan and lifespan.

Transcriptional control of inflammatory genes is achieved by multiple mechanisms, including three classes of transcription factors (NF-κB, AP-1, and STAT-1), various transcriptional co-regulators and chromatin modifications [57]. What are the mechanisms by which PHF2 regulates inflammatory gene expression in AD? One possibility is that PHF2 elevates the transcription of these genes by removing repressive H3K9me2 [33, 34, 37]. Alternatively, PHF2 is recruited to specific gene promoters to erase the repression checkpoint H4K20me3, facilitating their expression [35]. We found that Phf2 occupies the same loci as H3K4me3, but not H3K9me2, at the promoter region of inflammatory genes. The increased H3K4me3 occupancy in 5xFAD mice with increased Phf2 expression and the decreased H3K4me3 occupancy by Phf2 knockdown suggest that Phf2 might regulate inflammatory genes via a mechanism involving H3K4me3. Our findings add to the growing number of epigenetic readers and writers involved in regulation of neuroinflammation, a hallmark of AD.

The observed upregulation of PHF2 in AD and its regulatory effects on inflammation-related genes provide new insights into the molecular mechanisms underlying AD pathology. The connection between PHF2 activity and neuroinflammatory gene expression suggests that PHF2 could be a pivotal factor in the neuroinflammatory processes that contribute to neuronal damage and cognitive decline in AD. By regulating the expression of key inflammatory genes, PHF2 may shape the inflammatory environment of the AD brain. Elevated levels of cytokines and chemokines promote the proliferation and reactivation of microglia and astrocytes, which, in turn, release even greater amounts of the inflammatory mediators. Activated glial cells can directly contribute to the impairment of synaptic transmission and plasticity through an array of secreted and contact-dependent signals [58]. This disruption of neuron-glia signaling and synaptic function may significantly influence the progression of cognitive decline in AD.

In agreement with this, we have demonstrated that PHF2 knockdown in the 5xFAD mouse model leads to the reduction of microglia/astrocyte activation, restoration of synaptic function, and improvement of spatial memory. As the sEPSC amplitude and frequency are mainly determined by postsynaptic receptor numbers and presynaptic transmitter release probability, respectively, the rescue of sEPSC amplitude following Phf2 knockdown could be due to the reduced loss of postsynaptic spines because of inactivation of astrocyte/microglia. The differential effects of Phf2 knockdown in PFC on Barnes maze and NOR test are probably related to different neural circuits involved in these behavioral assays. PFC interacts with many subcortical and cortical regions to regulate a variety of self-regulatory and goal-oriented behaviors [59]. Phf2 knockdown in PFC may not be sufficient to restore the PFC network on which NOR depends. The results demonstrate the limitation of behavioral rescue when targeting one molecule in one brain region for a complex brain disorder.

The present study has identified PHF2 as a promising player regulating inflammation genes and ensuing biological processes in Alzheimer’s disease. PHF2 down-regulation in 5xFAD mice reduces the inflammation genes, which leads to the suppression of microglia/astrocyte reactivation. Consequently, the excessive loss of synapses (phagocytosis) was reduced, leading to the amelioration of synaptic and cognitive impairment. A cell type-specific knockdown approach will reveal whether the upregulation of Phf2 in neurons or in glial cells (e.g., microglia or astrocytes) plays a more critical role in neuroinflammation relevant to AD pathophysiology. Moreover, examining the effects of Phf2 knockdown in another AD model with tau pathology will confirm the findings’ general applicability. RNA-seq data of different brain regions have found the increased PHF2 in AD, suggesting that our results may not be restricted to PFC. However, detailed interrogation of other regions with pronounced AD pathology, such as the temporal lobes and hippocampus, awaits to be further studied.

### Experimental procedures

#### Ethics approval

All animal studies were conducted with the approval of the Institutional Animal Care and Use Committee (IACUC) at the State University of New York at Buffalo (IACUC ID: PROTO202000049). All methods were performed in accordance with the relevant guidelines and regulations.

#### Animals and human postmortem tissue

Transgenic mice carrying five familial AD mutations on human amyloid precursor protein (Swedish K670N/M671L Florida I716V and London V717I) and human presenilin1 (M146L and L286V) were maintained and genotyped as we previously reported [16–18]. Mice were group-housed (temperature: 72 °F; humidity: 56%) with *ad libitum* food accessibility in the 12-hr light-dark cycle (light: 6 am–6 pm; dark: 6 pm–6 am). Both male and female 5xFAD mice (6–7 months old) were used, and data were pooled together because of similarity.

Frozen human postmortem tissues (Brodmann area 10) from patients with AD and normal control subjects were provided by NIH NeuroBioBank (Supplementary Table 6). Upon arrival, tissue was stored in a −80 °C freezer until used for RNA extraction.

#### Generation of human iPSC-derived neurons

Human induced pluripotent stem cells (iPSCs) from both normal individuals (CW70305, CW70344, CW50040) and sporadic Alzheimer’s disease patients (CW50018, CW50024, CW50170) were acquired from the Human Pluripotent Stem Cell Line Repository at the California Institute for Regenerative Medicine (CIRM). These iPSCs were created using non-integrating Episomal vectors. All iPSCs were cultured on gamma-irradiated CF-1 mouse embryonic fibroblasts in a medium composed of DMEM/F12, 20% knockout serum replacement, 0.1 mM β-mercaptoethanol, 1× NEAA, 1× L-glutamine, and 4–8 ng/mL FGF2. For differentiation [60], iPSCs were dissociated using dispase to form embryoid bodies (EBs) and cultured in suspension in a (1:1) mixture of DMEM/F12 and Neurobasal media supplemented with N2 (1:100), B27 (without vitamin A, 1:50), NEAA, ascorbic acid (0.2 mM), SB431542 (10 µM, APExBIO), dorsomorphin dihydrochloride (5 µM, APExBIO), XAV939 (2.5 µM, APExBIO), and cyclopamine (3.5 µM, APExBIO). On day 6, the EBs were plated on Matrigel-coated plates and cultured until day 12 with media changes every other day. On day 12, dorsal cortex progenitors were dissociated into single cells using 1 unit/mL Accutase at 37 °C and plated onto polyornithine/Matrigel-coated plates at a density of 5000–10,000 cells/cm^2^ in DMEM/F12 and Neurobasal (1:1) media containing N2, B27 (without vitamin A, 1:50), 1× NEAA, ascorbic acid (0.2 mM), ROCK inhibitor Y27632 (20 µM) was added for the first 24 h. On day 16, cells were dissociated and replated in the same medium. At day 18, the medium was switched to Neurobasal containing B27 (without vitamin A, 1:50), Brain-derived Neurotrophic Factor (BDNF, 20 ng/mL), Glial cell line-derived Neurotrophic Factor (GDNF, 20 ng/mL), dibutyryl-cAMP (dcAMP, 0.25 mM), ascorbic acid (0.2 mM), and DAPT (2.5 µM), for the generation of cortical neurons. Half of the medium was changed every other day. Cortical neurons were cultured in this maturation medium until experiments were conducted at Day 40.

#### Bioinformatic analyses

Search for master regulators of AD DEGs were performed with TOPPGENE (https://toppgene.cchmc.org/prioritization.jsp) feature Transcription Factor Binding Site. Top 2000 AD DEGs ranked by transcriptomics [39] was used for the search. Phf2 gene expression analyses in microarray datasets were performed with AD concensus transcriptomics platform (http://swaruplab.bio.uci.edu:3838/bulkRNA/Swarup) [61]. Gene ontology pathway and process enrichment analysis was performed with Metascape (https://metascape.org/, mean overlap: 3; p value cutoff: 0.01; minimal enrichment: 1.5) [39, 40]. Gene network analysis was performed with STRING (https://string-db.org/) and Cytoscape, with hubs defined as nodes with >25 connecting edges. PHF2 target genes were retrieved using ChIPseq datasets SRR2932359 and SRR2932360 (parameters: −1kb to +100 bp of transcription start sites; sharp peaks: qvalue = 0.1, extsize = 200, mfold =  5, 50 bw = 500; broad peaks: qvalue = 0.1, broad-cutoff = 0.10, mfold = 5, 50 bw = 500). ChIPseq landscape was generated with IGV (https://igv.org/app/). Inflammation gene set was compiled based on Immunome Knowledge Base (Hs) [62]. Expression heatmaps were generated with Phantasus (https://artyomovlab.wustl.edu/phantasus/) using GEO accessions: GSE168137, GSE44770, GSE226901 and the count file generated from bioproject PRHJNA720779.

Residualized counts (derived from CQN normalized counts) from the RNAseq Harmonization Study (syn21241740) of 3 datasets (Mayo, MSBB, ROSMAP) were obtained using the following python (v. 3.13+) packages: pandas (v. 2.2.3) and numPy (v. 2.2.0) were used to load and modify the dataset matrices containing residualized counts of selected brain regions and conditions. Non-parametric hypothesis testing was implemented using scipy (v. 1.15.2) and scikit-posthocs (v. 0.11.3). Kruskal-Wallis tests assessed global expression differences across diagnostic groups (α = 0.05), with significant results followed by Dunn’s *post hoc* pairwise comparisons. False discovery rate (FDR) correction was applied to Dunn’s p-values using the Benjamini-Hochberg method (stats models v. 0.14.4). Analyses were performed separately for male, female, and combined cohorts to evaluate sex-specific effects. Multi-panel violin-boxplot composites were generated with seaborn (v. 0.13.2) and matplotlib (v. 3.10.1), featuring violin distributions (kernel density estimates), superimposed boxplots (IQR/median) and strip plots (individual samples), brain region-specific jittering, annotated significance bars (***p < 0.001; **p < 0.01; *p < 0.05), sex-stratified sample sizes and Kruskal-Wallis p-values.

#### Generation of Phf2 knockdown or overexpression reagents

Short-hairpin RNA (shRNA) sequences targeting mouse Phf2 (Supplementary Table 7) were obtained from Millipore Sigma Predesigned shRNA (https://www.sigmaaldrich.com/US/en/semi-configurators/shrna?activeLink=productSearch). The shRNA was cloned into a GFP-tagged adeno-associated virus (AAV) vector under the control of the U6 promoter (Addgene, Cat. #85741). AAV viral particles were produced by the viral core center at Emory University.

Single guide RNA (sgRNA) sequences (Supplementary Table 7) were designed using ChopChop (https://chopchop.cbu.uib.no/). Annealed sgRNA oligos were cloned into a *Bsm*BI-digested expression plasmid (Addgene, Plasmid #138461), where sgRNA transcription is driven by the U6 promoter.

Neuro-2a (N2A) cells (ATCC® CCL-131™) were cultured in DMEM medium (Gibco, 11995065) supplemented with 10% heat-inactivated fetal bovine serum (Gibco, 10438026) and 1× penicillin-streptomycin (Gibco, 15140122). Plasmids were transfected into 65–70% confluent mouse N2a cells using Lipofectamine 3000 (Invitrogen). For Phf2 overexpression experiments, each sgRNA plasmid was co-transfected with a dCas9-p300 expressing plasmid, pLV-dCas9-p300-P2A-PuroR (Addgene, Plasmid #83889). Transfection with sgRNA alone was used as the control. RNA was extracted 48 h post-transfection for qPCR analysis.

#### Animal surgeries

Mice received bilateral stereotaxic injections of AAV9 vectors (shRNA-AAV-GFP, 1.0 μl per hemisphere) into the medial PFC as previously described [17, 63–65]. Briefly, mice were anesthetized and placed on a stereotaxic apparatus (David Kopf Instruments). Injections were performed with a Hamilton syringe (needle gauge 31) at a rate of 0.1 μl/min, with the needle remaining in place for an additional 10 min. The virus was delivered to the target area using the following coordinates: 2.0 mm anterior to bregma, 0.25 mm lateral, and 2.25 mm dorsal-ventral. All experiments were conducted 10–20 days post-surgery.

#### Quantitative real-time PCR

Quantitative PCR was performed as previously described [16, 63]. For mice, fresh brain PFC samples were quickly homogenized in TRIzol and RNA was extracted following the manufacturer’s protocol (Cat# 5596026, ThermoFisher Scientific). Human postmortem samples were removed in cold room from tissues stored in −80 °C freezer. Synthesis of cDNA was performed with iScript cDNA Synthesis Kits (BioRad). Quantitative real-time PCR was performed on CFX Connect real-time system using the iQ SYBR Green Supermix (BioRad). GAPDH or Gapdh was used as the internal control. A total reaction mixture of 20 µl was amplified in a 96-well thin-wall PCR plate (Bio-Rad) using the following cycling parameters: 95 °C for 5 min followed by 40 cycles of 95 °C for 30 s, 58 °C for 30 s, and 72 °C for 60 s. Primers used in this study are shown in Supplementary Table 7.

#### Western blotting

For Western blotting of total proteins, iPSC-derived neuronal cultures at D40 were washed three times in cold PBS buffer and lysed in 1% SDS with protease inhibitor for 10 min at room temperature. Lysates were boiled for 5 min at 100 °C and centrifuged at 13,000 g for 10 min. Supernatant containing total proteins were separated on sodium dodecyl sulfate (SDS)–polyacrylamide gels and analyzed by Western blotting with antibody against PHF2 (Invitrogen, #PA5-35949) or GAPDH (Proteintech, #60004-1-1 g). ECL detection was performed according to the manufacturer’s protocol via SuperSignal™ West Femto Maximum Sensitivity Substrate (Amersham, Piscataway, NJ).

For Western blotting of nuclear proteins, N2A cells cultured in 12-well plates were collected in PBS and washed twice. The cell pellets were then resuspended in 1× hypotonic buffer (20 mM Tris-HCl, pH 7.4, 10 mM NaCl, 3 mM MgCl_2_, 0.5% NP-40), supplemented with 1 mM PMSF and a protease inhibitor cocktail to disrupt the cell membrane by pipetting up and down six times, and incubated on ice for 15 min. The homogenate was centrifuged at 3000 rpm for 10 min at 4 °C. The pellet was thoroughly resuspended in 1% SDS and then boiled for 5 min. After a brief centrifugation, the supernatant was used for quantification using the BCA protein assay kit (Pierce™). A total of 20 µg of protein from each sample was heated in loading buffer and loaded onto a 10% SDS-PAGE gel for electrophoresis. Proteins were transferred onto nitrocellulose membrane using the Trans-Blot Turbo Transfer System (Bio-Rad). Phf2 antibody (Invitrogen, # PA5-35949) and H3 antibody (# 701517) were used as primary antibodies. Proteins were detected using either an anti-mouse (1:2000, GE Lifesciences, NA931) or anti-rabbit (1:2000, GE Lifesciences, NA934) secondary antibody IgG coupled to peroxidase, and developed using ECL (SuperSignal West-Pico, Thermo Fisher, Waltham, MA, USA). Images and data were acquired using the ChemiDoc XRS system (Bio-Rad). Data analysis was performed using ImageJ software.

#### ChIP assays

ChIP-qPCR followed the procedures as what we described previously [16, 63]. N2A cells (48 h after transfection) or 5xFAD PFC tissues were collected in 250 μl ice-cold douncing buffer (10 mM Tris-HCl, pH 7.5, 4 mM MgCl_2_, 1 mM CaCl_2_) and homogenized using a 26-gauge syringe. The homogenized sample was incubated with micrococcal nuclease (5 U/ml) for 7 min at 37 °C, and the reaction was terminated with EDTA (10 mM). The sample was then incubated with 0.4 ml hypotonic lysis buffer (with protease inhibitors) on ice for 1 h, vortexing briefly every 10 min. After centrifugation at 3000 x g for 5 min at 4 °C, the supernatant was collected. A fraction of the supernatant (2%) was saved as the input control, and the remaining supernatant was pre-cleared with protein A agarose slurry for 1 h at 4 °C. Pre-cleared supernatant was incubated with the antibody (4 μg) against Phf2 (Invitrogen, #PA5-114542) or H3K4me3 (Invitrogen, #PA5-3594) overnight at 4 °C, followed by incubation with agarose slurry for 2 h. After washing with low salt, high salt, LiCl wash buffer, and TE buffer, the bound complex was eluted with elution buffer (1% SDS, 0.1 M NaHCO_3_) at room temperature. Eluates and inputs were treated to remove protein and RNA in EDTA (0.5 M), NaCl (5 M), Tris-HCl (10 mM, pH 7.4), RNaseA (10 mg/ml), and proteinase K (20 mg/ml) and incubated at 60 °C for 1 h. DNA was purified with isopropanol and desalted with 70% ethanol or purified by QIAquick PCR purification Kit (Qiagen, 28104) and finally dissolved in EB buffer. ChIP-qPCR primers targeting *Notch2* or *Nfkbia* promoter are included in Supplementary Table 7. ChIP signals were quantified as % input.

#### Immunohistochemistry

Mice were anesthetized and transcardially perfused with PBS, followed by 4% paraformaldehyde (PFA) prior to brain removal. Brains were post-fixed in 4% PFA for 24 h, then treated with 30% sucrose for 3 days. Subsequently, the fixed brains were sectioned into 50 μm coronal slices. The slices were washed three times in PBS, then blocked for 1 h in PBS containing 5% Bovine serum albumin (BSA) and 0.2% Triton X-100 for permeabilization. After washing, the slices were incubated with primary antibodies with gentle shaking for overnight at 4 °C. The primary antibodies used were Iba1 (1:500, 019-19741, Wako Chemicals, Richmond, VA), CD68 (1:500, 137001, BioLegend, San Diego, CA), GFAP (1:500, PA5-85109, Invitrogen), and NeuN (1:500, MAB377, Millipore Sigma). Following three washes in PBS (10 min each with gentle shaking), the slices were incubated with secondary antibodies (1:1000 dilution) for 1.5 h at room temperature, followed by three additional PBS washes. The secondary antibodies used included AlexaFluor 594 goat anti-rabbit (A11037) and AlexaFluor 647 goat anti-mouse (A-21235) from Invitrogen. Slices were mounted on slides with Vectashield mounting medium with DAPI (Vector Laboratories) or ProLong Diamond Antifade mounting medium with DAPI (Invitrogen). Images were acquired using a Leica TCS SP8 confocal microscopy system. All specimens were imaged under identical conditions and analyzed using Fiji-ImageJ software with identical parameters.

#### Electrophysiological recording

Whole-cell voltage-clamp recording was employed to measure synaptic currents in PFC layer V pyramidal neurons. PFC-containing mouse brain slice (300 µm thick) was placed in a perfusion chamber attached to the fixed stage of an upright microscope (Olympus) and submerged in continuously flowing oxygenated artificial cerebrospinal fluid (ACSF) containing (in mM): 130 NaCl, 26 NaHCO_3_, 1 CaCl_2_, 5 MgCl_2_, 3 KCl, 1.25 NaH_2_PO_4_, and 10 glucose, pH 7.4, 300 mOsm. For EPSC recordings, bicuculline (10 µM) was added to the ACSF. The Electrode internal solution consisted of (in mM): 130 cesium-methanesulfonate, 10 CsCl, 4 NaCl, 10 HEPES, 1 MgCl_2_, 5 EGTA, 2 QX-314, 12 phosphocreatine, 5 MgATP, 0.5 Na_3_GTP, and 0.1 leupeptin, pH 7.2–7.3, 265–270 mOsm. Spontaneous EPSC were recorded at −70 mV holding potential. Evoked EPSC was recorded with a bipolar stimulating electrode (FHC, Bowdoinham, ME) positioned ~100 μm from the recorded neuron. The stimulating electrode was controlled by an S48 stimulator (Grass Technologies, West Warwick, RI). Stimulation pulses (0.06 ms, 6 V) were delivered at a frequency of 0.05 Hz. AMPAR-EPSC was recorded at −70 mV, followed by the recording of mixed AMPAR- and NMDAR-EPSC at +40 mV using the same stimulation parameters. The peak of NMDAR-EPSC was determined 40 ms after the onset of mixed EPSC. Data were analyzed using Clampfit 10.0.7 software (Molecular Devices, Sunnyvale, CA).

#### Behavioral testing

The investigator was blinded to the group allocation during the experiments and the outcome assessment. Barnes maze tests were performed as previously described [16, 17, 19]. Briefly, the mouse was placed on a round platform with eight equally spaced holes at the edge, one of which was attached with an escape box (correct hole). Bright overhead light was applied as a weak aversive stimulation to increase the motivation to escape from the circular platform. The mouse was first allowed to habituate to the platform for 5 min. During the two learning phases for information acquisition (5-min interval), the mouse was allowed to explore the platform using distal visual cues until finding the correct hole and entering the escape box. Then, the mouse was placed in its home cage for 15 min. In the memory phase (information retention and retrieval), the escape box was removed, and the mouse was put back on the platform to explore for 5 min. The time spent investigating the correct hole (T_1_), the other seven incorrect holes (T_2_), and the latency of reaching the correct hole for the first time (L) were counted. Spatial memory index was calculated by ((T_1_-T_2_/7)/(T_1_+T_2_/7))/Log_2_(L). The pre-injection tests were performed at ~6 months old and post-injection tests were performed at ~7 months old. Because of the long gap between tests, mice were re-trained to find the correct location before testing the memory phase. Our previous studies have confirmed the validity of repeated measurements in Barnes maze tests [16, 17, 19].

Novel object recognition tests were performed as previously described [16, 17, 19]. Briefly, after habituation, the animal was placed on a circular open platform (2 feet in diameter) and allowed to explore two identical objects for 5 min before being returned to its home cage. After a 5 min interval, the mouse was placed back on the platform, where one of the original objects remained (familiar), while the other was replaced with a new object (novel). The mouse’s interactions with each object were recorded for 5 min. The discrimination ratio was determined using the formula: (Time on novel object – Time on familiar object) / (Time on novel object + Time on familiar object).

#### Statistical analysis

Statistical analyses were mainly performed using GraphPad Prism. Hypergeometric analysis was conducted with the Graeber lab online calculator. Experiments involving two groups were analyzed using two-tailed unpaired t-tests unless stated otherwise. For experiments with more than two groups, one-way ANOVA with Bonferroni correction for multiple *post hoc* comparisons was applied. Data were tested for normality prior to parametric analysis. The variance was similar between the groups that were statistically compared. All data points represent distinct samples and are presented as the mean ± SEM. Data points identified as statistically significant outliers (determined by Grubb’s test, *p* < 0.05) were excluded from the analyses. Sample sizes were determined based on power analyses and were comparable to those reported in previous studies.

## Supplementary information

Supplementary Figures

Supplementary Tables

## Data Availability

The accession numbers of data deposited to public databases have been included in the main text. All other data are available upon requests.
