# Supplementary material for: Histone demethylase PHF2 regulates inflammatory genes in Alzheimer’s disease
Source: Mol Psychiatry. 2025 Aug 23;31(2):845–59. doi: 10.1038/s41380-025-03181-z (PMC12746590; doi:10.1038/s41380-025-03181-z)
Supplement: Supplementary file 1 — Supplementary Figures [file 41380_2025_3181_MOESM1_ESM.pdf]

Histone Demethylase PHF2 Regulates Inflammatory Genes in Alzheimer's Disease

Guojun Yang, Yong Ren, Ping Zhong, Prechetas Jai Patel, Xiao-Qing Chen, Lei Wan, Young-Ho Lee, Komal Saleem, Jian Feng, and Zhen Yan*

Supplementary Figures:

Sup. Figure 1. PHF2 mRNA is increased in AD patients from 3 bulk RNAseq datasets. A-C, Box and Violin plots of residualized counts of PHF2 from Harmonization of 3 datasets: Mayo (A), temporal cortex (n=147); MSBB (B), frontal pole and inferior frontal gyrus (n=398); and ROSMAP (C), dorsolateral prefrontal cortex and posterior cingulate cortex (n=675). The comparisons for each dataset were subdivided into males only, females only and combined sexes, further subdivided into 3 conditions: control (Braak stage $\leqslant$ 2), Moderate AD (Braak stage 3-4), and Severe AD (Braak stage $\geqslant$ 4). Kruskal-Wallis and *post hoc* Dunn tests were performed to test statistical significance (p-values listed on top of each plot, significance marked with *).

Sup. Figure 2. Phf2 knockdown in 5xFAD mice does not alter the expression of most of the selected synaptic genes. A, Bar graphs of qPCR data showing mRNA levels of selected synaptic genes in PFC of 5xFAD mice injected with GFP control (Con) or Phf2 shRNA (shPhf2) AAV (n=10/group). Data are presented as mean ± SEM. *$p < 0.05$, unpaired t-test.

Supplementary Tables:

Sup. Table 1: Enrichment of transcription factor binding sites in AD DEGs (for Fig. 1A)

Sup. Table 2: PHF2 expression levels in control vs. AD humans (for Fig. 1B)

Sup. Table 3: Statistics of PHF2 changes in different stages and sexes of AD (for Sup. Fig. 1)

Sup. Table 4: PHF2 changes in scRNA-seq of AD (for Fig. 1C)

Sup. Table 5: Gene sets for GO enrichment and gene network analyses (for Fig. 2A-C)

Sup. Table 6: Metadata for human postmortem samples

Sup. Table 7: Oligos used in qPCR, shRNA, sgRNA and ChIP

Fig. S1

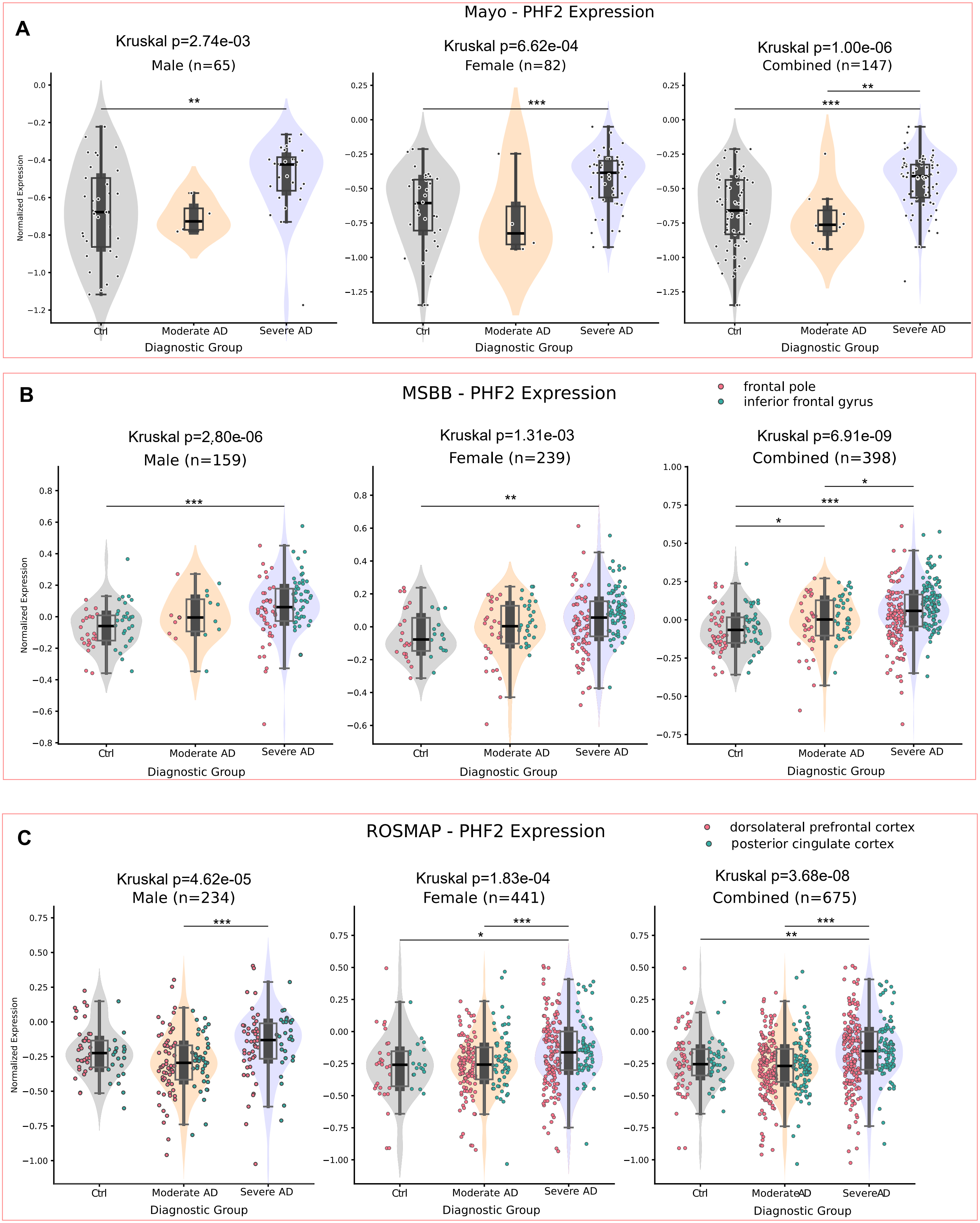
A
Mayo - PHF2 Expression
Kruskal p=2.74e-03
Male (n=65)
Kruskal p=6.62e-04
Female (n=82)
Kruskal p=1.00e-06
Combined (n=147)
Normalized Expression
Ctrl
Moderate AD
Severe AD
Diagnostic Group
B
MSBB - PHF2 Expression
frontal pole
inferior frontal gyrus
Kruskal p=2.80e-06
Male (n=159)
Kruskal p=1.31e-03
Female (n=239)
Kruskal p=6.91e-09
Combined (n=398)
C
ROSMAP - PHF2 Expression
dorsolateral prefrontal cortex
posterior cingulate cortex
Kruskal p=4.62e-05
Male (n=234)
Kruskal p=1.83e-04
Female (n=441)
Kruskal p=3.68e-08
Combined (n=675)

Fig. S2

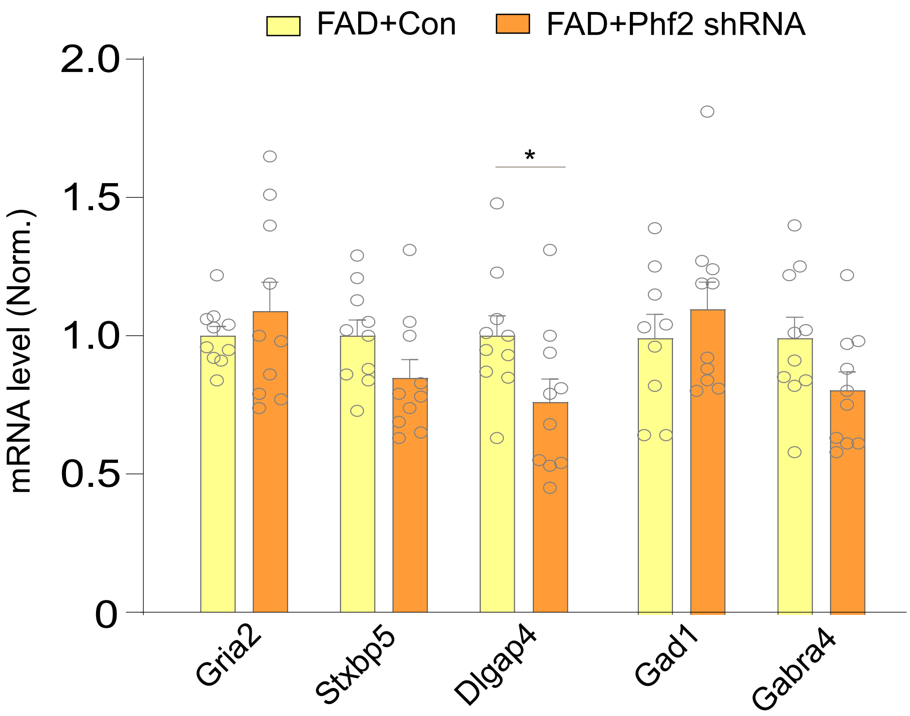

FAD+Con
FAD+Phf2 shRNA
mRNA level (Norm.)
2.0
1.5
1.0
0.5
0
*
Gria2
Stxbp5
Dlgap4
Gad1
Gabra4
